# Exploring the barriers to mental health help-seeking among African Migrants in Australia: A qualitative study

**DOI:** 10.1177/00207640251323050

**Published:** 2025-03-18

**Authors:** Nnaemeka Meribe, Obed Adonteng-Kissi, Kathomi Gatwiri, Lillian Mwanri, Frank Darkwa Baffour, Agness Tembo, Edith N. Botchway-Commey, David Chisanga, Ahmed A. Moustafa, Kerrie E. Doyle, Uchechukwu Levi Osuagwu

**Affiliations:** 1 La Trobe University - Melbourne Bundoora Campus, VIC, Australia; 2 Edith Cowan University, Bunbury, WA, Australia; 3 Southern Cross University, Lismore, NSW, Australia; 4 Torrens University Australia, Adelaide, SA, Australia; 5 University of Newcastle Family Action Centre, Callaghan, NSW, Australia; 6 The University of Sydney, NSW, Australia; 7 University of Melbourne, Parkville, VIC, Australia; 8 La Trobe University, Melbourne, VIC, Australia; 9 Bond University Ltd, Gold, QLD, Australia; 10 Western Sydney University, Campbeltown, NSW, Australia; 11 School of Medicine, Western Sydney University, Campbelltown, NSW, Australia

**Keywords:** Mental health, help-seeking, barriers, African migrants, religion, cultural beliefs

## Abstract

**Background:**

The health of African migrants in Australia is a largely under-researched topic despite the steadily increasing size of the population and its uniqueness. In particular, few studies have explored the mental health of African migrants in Australia or their utilization of mental health services.

**Aims:**

This study explored the barriers to mental health help-seeking among first-generation African migrants in Australia.

**Methods:**

In this qualitative study conducted using the hermeneutical phenomenological research approach, the purposive sampling method was used to recruit participants. In-depth interviews with participants were undertaken online via Zoom, Teams, and WhatsApp calls. Interviews were recorded and transcribed verbatim, utilizing a thematic analysis as the primary data analysis method.

**Results:**

African migrants were more inclined to seek support for mental health conditions from religious figures such as priests instead of seeking professional help. Religion and poor knowledge about mental illness were highlighted as barriers to mental health help-seeking. Participants also considered cultural beliefs, fear of stigma as well as the high cost of healthcare in Australia as significant barriers to mental health help-seeking among African migrants.

**Conclusions:**

Findings reinforce the critical need for culturally competent mental health services tailored to the beliefs, values, religion, and experiences of African and other migrant communities in Australia. Given the strong attachment of many African migrants in Australia to their cultural and religious beliefs, such services are essential for practical support and intervention.

## Introduction

In recent years, research on the mental health of international migrants has begun to garner scholarly attention. The significant increase in global migration in the past two decades has directed attention to the impact of migration on individuals, societies, and healthcare systems. Researchers are beginning to recognize the importance of the unique health challenges that international migrants face while relocating to another country and adapting to a new culture and unfamiliar social terrain. Adjustment to life in a new land by many new migrants poses various difficulties; for instance, adjusting to a different culture and societal norms, learning a new language, coping with economic challenges, and having smaller family and community networks. Migration poses distinct difficulties for parents and young people. Varied levels of acculturation (Taket et al., 2009) between older family members and young migrants, with the young population adjusting to situations at a quicker rate, may heighten family stress and lead to substance use and mental health challenges (Abood et al., 2021; Taket et al., 2009).

Australia ranks among the top 10 destinations for international migrants (Australian Bureau of Statistics, 2023a). More than a quarter (27.7%) of the country’s population are foreign-born (Australian Bureau of Statistics, 2023a), indicating that migrants constitute a significant proportion of Australia’s population. In 2023, migrant arrivals in Australia increased 73% to 737,000 from 427,000 arrivals the previous year (Australian Bureau of Statistics, 2023b). An increase in migration results in demographic changes, underscoring the need to provide effective mental health care for culturally diverse populations (Jarvis et al., 2020). Hence the United States’ Center for Substance Abuse Treatment (2016) admonishes mental health professionals to consider the effects of culture when diagnosing clients. Mental health professionals working with culturally diverse patients can improve their cultural competence by utilizing the cultural formation interview (CFI), a systematic approach developed by the American Psychiatric Association (APA) for mental health professionals working with culturally diverse patients and published in DSM-5 (American Psychiatric Association [APA], 2013).

This paper, however, focuses primarily on African migrants in Australia, a sub-population that has proliferated lately. From the 317,183 people who identified as African-born^1^ in the 2016 Census (Apat & Digwa, 2021), the population of African migrants in Australia rose to about 400,000 in 2020, representing 1.6% of the Australian population and 5.1% of Australia’s overseas-born population (Counted & Renzaho, 2021). Most of the population arrived in Australia as humanitarian entrants, skilled migrants, and students through family stream visa programs (Apat & Digwa, 2021), indicating a highly diverse group with potentially varying levels of mental health awareness and engagement.

Political and economic instability and civil wars across Africa in the recent past forced millions of Africans to migrate to more stable regions. War, violence and famine are serious traumatic events that cause different kinds of injuries, fear, unsafety, and hopelessness – and this is the initial pre-migration stress faced by migrants, including African migrants (Boskovic & Jankovic, 2023). African migrants also face pre- and post-migration traumas and stressors that shape their experiences and heighten their vulnerability to mental health challenges such as PTSD, depression, and anxiety (Botchway-Commey et al., 2024; Tessitore et al., 2022). However, research indicates that only a few migrants seek professional help for their mental health challenges (Krystallidou et al., 2024; Mohammadifirouzeh et al., 2023). This is problematic, as help-seeking delay can adversely affect social, educational, and vocational outcomes (Clement et al., 2015; Marshall et al., 2020), leading to lengthy periods of poorer outcomes and untreated illnesses (Scott et al., 2022). While migrants are not a homogenous group in the context of help-seeking for mental health challenges, help-seeking delays are common in these groups (Byrow et al., 2020; McCann et al., 2016). This is confirmed in a Netherlands study, which finds that undocumented migrants are more expected to seek help from informal help sources, such as family, friends and religious institutions, than seek professional help (Vollebregt et al., 2023).

Help-seeking behaviors among migrant groups are influenced by many factors, even though for alcohol, drugs, and mental health challenges (McCann et al., 2016; Schubert et al., 2019), inadequate mental health literacy is especially paramount (Kutcher et al., 2016). The varied understanding and conceptualization of mental health, alcohol and other drug challenges associated with inadequate awareness of treatment options and services are expected to negatively affect help-seeking (Dowman, 2017; Pretorius et al., 2019). An Australian study conducted in eight migrant communities in Victoria recognized several help-seeking barriers: expectations and needs of clients as well as of the diversity of people accessing their services; services’ insufficient knowledge of the perceptions; lack of advocacy by community leaders; language barriers; a desire to have the capacity to deal with alcohol and drug problems; community pressures to be discreet about drug use; and lack of family inclusion in alcohol and drug programs (McCann et al., 2016).

The health of African migrants in Australia is a largely under-researched topic despite the steadily increasing size of the population and its uniqueness. Correspondingly, few studies have explored the mental health of African migrants in Australia or their utilization of mental health services (Apat & Digwa, 2021; Fauk et al., 2021, 2022; McCann et al., 2017; Mwanri et al., 2022; Omar et al., 2017) have explored the mental health of African migrants in Australia or their utilization of mental health services. Although most studies focus on individuals with refugee backgrounds and asylum seekers (Abur & Mphande, 2019; Fauk et al., 2021; McCann et al., 2016; Omar et al., 2017), many of the African migrants arrived in Australia as voluntary migrants (Apat & Digwa, 2021). Therefore, the present study seeks to expand the current literature by exploring the barriers to mental health help-seeking among first-generation African migrants in Australia.

## Theoretical Framework

Access to health care has been a public health issue in developed and developing countries (Cu et al., 2021). Timely access to quality health care is critical to preventing, treating, and managing diseases (Australian Institute of Health and Welfare (AIHW, 2020). Individuals or groups face barriers to healthcare when they are unable to access timely and appropriate health care (AIHW, 2020). These barriers can cause impairment to health if they remain unaddressed Access to healthcare is an intricate concept closely linked with health systems performance (Levesque et al., 2013). Access to healthcare entails being able to reach and obtain the right healthcare services when there is a perceived need for care (Levesque et al., 2013, p. 4). Accessing healthcare, including mental healthcare, is one of the biggest challenges faced by migrants in their host countries (Buja et al., 2013). Insufficient access to healthcare is a significant post-migration stressor that leads to progressive deterioration of health (Marmot et al., 2008). This is worsened by the paucity of information and support, which can affect an individual’s decision to seek help or treatment (National Collaborating Center for Mental Health, 2011).

The Conceptual Framework of Access to Health offers an exciting and comprehensive model for analyzing access to healthcare. The framework views access to health care services as the outcome of the intricate interface between the quality of the services, service providers, health systems, and organizations on the one hand, and clients/patients and their environments on the other hand (Hynie et al., 2022). Introduced by Levesque et al. (2013), the framework offers a multidimensional view of healthcare access in the context of health systems, juxtaposing five dimensions of accessibility of the healthcare system, including approachability, acceptability, availability, and accommodation, affordability, and appropriateness; with patients’ abilities to identify health needs, seek, reach, and utilize care (Cu et al., 2021; Voorhees et al., 2021). The framework also incorporates a time element and presents each dimension sequentially to mirror the patient’s healthcare journey (Graetz et al., 2017).

The approachability dimension considers that individuals (migrants) can recognize that services exist, can be reached, and can impact their health. Approachability is influenced by transparency, information regarding treatments, and outreach activities are factors. Critical to this dimension is the population’s ability to perceive the need for care, which is determined by health literacy and health beliefs. Acceptability involves the cultural and social factors that determine the likelihood of people accepting aspects of the service (such as the sex or social group of providers, the cultural beliefs associated with systems of medicine) and the perceived appropriateness for the individuals to seek care (Levesque et al., 2013). Crucial to this dimension is the need to ensure that care providers cater to the needs of the disadvantaged in the population.

Availability entails the ease with which health services (either the physical space or those working in health care roles) can be reached physically and promptly (Levesque et al., 2013). This dimension considers the availability of the services and healthcare professionals adequately equipped to produce or deliver the services to individuals seeking help (Fauk et al., 2021). Access is limited if available resources are unevenly distributed across a country. Crucial to this dimension is people’s ability to reach the services. For example, the lack of transportation will impact the mobility of the aged and disabled. Affordability generally entails people’s financial capacity to access healthcare without significant economic challenges. It assesses people’s capacity to spend resources and time to use appropriate services (Levesque et al., 2013). According to Levesque et al. (2013), this dimension flows from direct prices of services and associated expenses as well as opportunity costs related to loss of income. However, this can vary by the nature of services and is based on the capacity to generate the resources to pay for care (Levesque et al., 2013). Appropriateness considers whether the services provided meet the healthcare needs of the population. It assesses how services fit the needs of individuals assessing the services. In this sense, access to low-quality services is considered a limitation of access to health care.

## Research Approach and Design

This paper was extracted from the qualitative component of a more extensive study that employed a sequential mixed methods research design to explore mental health help-seeking among African migrants in Australia. The qualitative research design is located within the interpretivist ontological paradigm (Dei-Anane et al., 2018). Interpretivism assumes that reality cannot exist without us knowing it (Grix, 2018), and, therefore, reality can best be understood by how people interpret their everyday social experiences in the world. Since the researchers were interested in the uniqueness of each participant’s story, a hermeneutical phenomenological approach was adopted for the qualitative component of the study. Hermeneutic phenomenology interprets experiences and phenomena through the individual’s lifeworld. This approach holds that a researcher must be aware of the influence of an individual’s background and accommodate the influences they exert on the individual’s experience of being (Neubauer et al., 2019). Drawing on this approach, this paper aimed to understand and shed light on the perceived barriers to mental health help-seeking amongst African migrants in Australia. As Cresswell (2013) explains, the qualitative researcher seeks to acquire a more thorough grasp of the phenomenon being studied.

### Recruitment

In the larger mixed-methods study, the researchers sent a participation invitation to leaders of selected African community groups/associations and organizations providing services for migrants to advertise on their websites and social media handles following permission. The participant information statement and the contact details of the field researchers formed part of the advertisement. A survey link was also created for the study using Research Electronic Data Capture (REDCap) to prevent non-Africans from completing it. The link was sent to heads of community associations for dissemination to all members through their database. The survey was also placed on online platforms (such as WhatsApp, LinkedIn, Twitter, and Facebook) to increase participation. Consent to participate was sought through a preamble in the survey, and only consenting eligible members were allowed to respond. To be eligible for the study, participants must be able to speak English, aged 18 to 59, born in Africa or as citizens of an African country at birth and have lived in Australia for at least 12 months. In addition, participants must either be mental health professionals or individuals with a career in mental health at the time of the study. Upon completion of the survey, participants were asked if they desired to be contacted for an interview. Those who indicated interest were contacted and purposively recruited for interviews. Overall, 12 participants were interviewed for the study. Cresswell (2013) considers a sample size of 12 participants adequate in phenomenological research. Each participant received a $25 supermarket voucher as reimbursement for their time. Participants were unaware of the gift voucher before signing up for the interview.

### Data Collection

We collected data via semi-structured interviews and an interview guide, as recommended by Naderifar et al. (2017). We found the semi-structured interview useful because it enabled us to explore participants’ thoughts deeper. Magaldi and Berler (2020) explain that although the semi-structured interview generally follows a guide or protocol, it allows the interviewer to explore beyond participants’ initial responses as the conversation unfolds. Each interview lasted, on average, 45 min. The interview guide was divided into three sections. The first section focused on questions for organizations or services, the second addressed individual mental health professionals, while the third contained questions for African migrants who were not mental health professionals. The guide contained 29 questions altogether, although some had follow-up questions. The interview method was explained in advance to the participants, and they were allowed to choose their preferred platform. The participants opted to be interviewed via Zoom, which they considered a convenient and secure medium for sharing their experiences without fear of being overheard by other people. Although the literature indicates that synchronous interviewing mode may affect the establishment of trust, as is the case with face-to-face interviews, because the use of a computer can be seen as a virtual barrier between the interviewer and the interviewee (Mann & Stewart, 2000), it was not seen as a limitation during the interviews for the present study. Indeed, conducting the interviews in a convenient, personal space facilitated openness among the participants. Through the interviews, the researchers explored the different barriers to mental health help-seeking among the participants. The interviews focused mainly on how participants felt about seeking professional help. Participants were also asked their views regarding whether seeking help from professionals could improve the condition of people with mental health issues. In addition to asking them what they considered as barriers to mental help-seeking within their communities, participants were further asked how their background as African migrants had influenced their views about mental health.

### Data Analysis

Interviews were audio-recorded with participants’ consent, transcribed verbatim by a professional transcriber and analyzed using thematic analysis (Castleberry & Nolen, 2018). Thematic analysis was conducted using Braun and Clarke’s (2006) framework analysis approach, where the researchers collectively agreed upon certain themes and sub-themes based on the study’s objectives. Initially, the researchers read the transcripts to understand each participant’s personal story and its meaning within the context of the research focus. Open coding was adopted to produce a preliminary set of codes following the reading and re-reading of the transcripts (Peel, 2020; Roberts et al., 2019). Furthermore, open coding was adopted because the researchers did not have pre-set codes and produced the codes as they read through the transcripts (Chang & Wang, 2021). Preliminary codes were scrutinized to identify original patterns and generate descriptive themes (Braun & Clarke, 2022). To ensure the themes accurately represented the interviewees’ comments, the researchers repeatedly analyzed and reviewed the themes (Roberts et al., 2019).

## Ethics

We obtained ethical approval from the Human Ethics Committee of the University of Western Sydney University, Australia (Approval No. H15116). Consistent with the University Ethics Committee guidelines, the researchers respected cultural differences, and the rights of participants were guaranteed before, during, and after the study. The voluntary nature of participating in the interviews was explained to participants before the commencement of the interviews. Each participant gave their informed consent before the interview was conducted. They were assured that their confidentiality would be protected and that data would be anonymized throughout the research process. Data gathered were anonymized as early as the transcription stage using pseudonyms, one of the strategies used by researchers to maintain participants’ confidentiality and anonymity (Farrugia, 2019).

### Trustworthiness

According to Lincoln and Guba (1985), the goal of trustworthiness in a qualitative inquiry is to reinforce the notion that paying attention to research findings is essential. Trustworthiness entails rigor in research and the level of confidence in data, interpretation, and methods employed to guarantee the integrity of a study (Pilot & Beck, 2014). Trustworthiness is critical to the usefulness and integrity of the findings (Cope, 2015). To ensure trustworthiness in this study, the researchers employed the criteria outlined by Lincoln and Guba (1985): credibility, dependability, confirmability, and transferability. Credibility was ensured through investigator triangulation, during which two or more team members collected and analyzed data for the study. The researchers also maintained self-awareness and stayed engaged throughout the research process (Probst, 2015). Self-knowledge helped to inform and enrich the research endeavor, and all the qualitative researchers on the larger research team are first-generation African migrants and thus understand the mental health struggles of African migrants. Self-reflection and feedback from the team leader, an experienced qualitative researcher, offered new layers of meaning. The researchers enhanced transferability by being trustworthy and transparent about analysis and providing a vivid picture that will inform and resonate with readers (Amankwaa, 2016). To ensure dependability, the researchers maintained an audit trail of process logs and engaged in peer debriefings with the team leader. In addition to preparing detailed drafts of the study protocol throughout the study, the researchers also kept a comprehensive record of the data collection process (Forero et al., 2018). For confirmability, the researchers digitally recorded the interviews with the participants’ permission and transcribed the data verbatim to avoid possible bias (Connelly, 2016).

## Results

Thematic analysis showed five overarching themes: Religion and deference to religious leaders, perceived poor knowledge about mental health, Perceived fear of stigma and cultural beliefs about mental health, Perceived cultural insensitivity and poor service delivery, and Cost of mental health services. The themes are presented below:

### Religion and Deference to Religious Leaders

Many participants reported that religion constituted a barrier to mental health help-seeking. They noted that many people in their communities were more likely to contact their religious leaders (priests) for mental disorders than seek professional help. For example, a participant, P4, said: ‘A lot of people try to contain things at home, or the church provides spiritual support to the mental health patient’. Another participant, P1, felt that religion was preventing African migrants from addressing the real issue:

We try to make everything about religion; we talk about going to church and finding God in that situation. It will not be addressed, so we leave everything to God. I am a Christian. So, we use religion to dampen what you are going through rather than address the issue.

Some participants suggested that poor understanding of mental health was the reason why African migrants sought help from religious leaders rather than mental health professionals:

Maybe they think, ‘Oh, somebody has been bewitched, demon-possessed, or some spiritual thing’. They go to the church for help with that, and yet it is a mental health issue rather than a spiritual issue. – P11.

People who have mental health issues think they’ve got demons. They have got devils. Most of them will bring them to church to pray for, or they have got spiritual attack. They associate that with spirituality, and people who -see them will say they’ve done something evil. It’s the result of what you’ve done, maybe, so spiritual. – P2

Like somebody. . .suffering from depression, instead of going to see a counsellor, they might decide to go to a priest to pray for them. . . They look at it from a religious point of view because they are not well-informed about mental health. – P7

Several of the participants consider religion as a barrier to mental health help-seeking. They understand that religion could influence people’s views regarding mental illness and whether – or not – they will seek help and where they will seek help.

### Perceived Poor Knowledge About Mental Health

Participants agreed that poor mental health knowledge and awareness were significant barriers to mental health help-seeking in African migrants. Some noted that there was a general perception that mental health was a Western concept, noting that many Africans in Australia were covering up their mental health challenges and appeared less interested in getting the required education:

Many of us think that mental health challenges are a Whiteman’s illness. It’s not our illness, so whenever I tell someone, "Oh, those look like signs of depression, they are like, I’m just stressed. That’s part of it. People don’t know what depression looks like. - P6

. . .Coming from an African perspective, we see it [mental illness] as, oh, it’s diabolical, or it’s spiritual. It’s not just an illness, no. You can’t suddenly lose your interest or don’t want to connect, or you stay on the wrong, or you start self-harm or things like that. No, it’s spiritual, or no, something is wrong. Something is wrong, really, but what is it? That’s the question. If we break past that, it gives us a different perspective. -P.11

One participant blamed poor knowledge of mental health on the African migrants’ inability to know when to take a break and attend to their mental health by seeking professional help:

I came here, and that was my perspective on things, so as I was experiencing my own challenges, I would say to myself, ‘Just keep moving. You get up, you keep going, you keep going’. Until there came a point where I went, you know what? I’m really tired, and as much as I want to keep pushing, there are these limitations now as to how much I can keep motivating myself to keep going as these issues compound or as I’m also evolving as a person. The biggest learning curve for me came when I had a personal experience with family violence, and that was a big turning point because I was lucky enough to, at the time, be able to speak up, and I had a wonderful GP who was helpful.– P5

The participant added that because many Africans had a poor understanding of mental health, they get sucked deeply into the rat race of life, piling more pressure on themselves until they get burnt out. At times, the pressure might come from family members both in Australia and back in their home countries who lack knowledge of mental health. Another participant, P10, stated that because many African migrants lacked adequate information about mental health, they were unable to identify early indicators of mental health issues.

### Perceived Fear of Stigma and Cultural Beliefs About Mental Health

All the participants indicated that fear of stigma and cultural beliefs constitute a significant barrier to mental health help-seeking among African migrants in Australia. For instance, P4 noted: ‘Yeah, sometimes African people with mental health problems will not want to talk about it because of the stigma. During the six months . . . I saw maybe three African migrant clients who came to seek help’. The participant added: ‘Even the African migrant clients that I saw were involuntary clients; they came in because they were forced to come and see me in the hospital’.

P9 explained that the stigmatization of mental illness was a product of African culture, which frowns upon any form of weak-mindedness:

. . . stigma comes from our culture, which requires that someone be consistently strong and capable of dealing with challenges and that certain things are not talked about. I think that’s where it comes from because there’s just an expectation that you have to be strong. . . It is certainly not talked about, perhaps as it should.

For P1, many African migrants ‘are still stuck from where they came from to Australia; they can’t see themselves going any further or changing’. Another participant, P4, related that African men were trained to be tough and strong and not to show emotions even in challenging times. Consequently, the participant explained seeking help would be seen as a sign of weakness. Even when they eventually agree to seek help, P4 added, they prefer professionals without African backgrounds who would not consider them weak:

African migrant men are reluctant to seek help from African migrant therapists/clinicians, whether men or women, because African migrants think their vulnerability will be shown before any Black African person. I remember a lot of Black African men said they prefer to see a White therapist/clinician or non-Black African therapist/clinician because it is too confronting, which is interesting.

Similarly, P5 explains that it is considered a shame within the African community for one to say that one is depressed:

If I had a terrible day and I’m feeling depressed and I stay in bed all day, it’s not something to speak about because it’s not expected. It’s shameful. . . That’s part of it as well. I also think it has to do with our own level of self-determination because of external factors.

P10, however, added that because the average African migrant in Australia did not want to be pitied or be seen as a ‘crazy person’ by people within their community, they would avoid any opportunity to see mental health professionals. The participant said, ‘If I were to access the services, that would be the biggest thing. Even now, even knowing what I know, you don’t want to be outed as a crazy person. I think the stigma is a big thing’.

### Perceived Cultural Insensitivity and Poor Service Delivery

A key barrier that the participants highlighted was cultural insensitivity. Many of the participants believed that many mental health service providers were not sensitive to the cultures of service users, a situation which they believe discourages many African migrants from seeking professional help for their mental health problems. Stressing the importance of having culturally sensitive service providers, one participant, P12, recalled how she felt safe and reassured after seeing a psychologist who had worked with service users from a Culturally and Linguistically Diverse (CALD) background:

. . .This psychologist had worked for many years in a community with a diverse migrant background, including Africans, Italians, Lebanese. . . While she wasn’t African, I could tell she understood what I was saying when I spoke about something. I was lucky to relate to a psychologist who was extremely helpful. We had a good connection from the start, and she really supported me through my journey in overcoming the challenges I was having at that time.

Another participant, P7, who was more particular about young people, felt that there was a need to have service providers who would understand that young African migrants face different challenges from others: ‘Most of the services provided, they’re not specific to African young people, and that’s the other thing that’s a challenge that the issues facing young African Australians are different to the issues facing other young Australians’.

Other responses from participants include:

I would prefer an African. The person needs to be relatable. Even in looking at someone, there’s power in talking to someone who looks like you. I’m more open to talking about things because I feel like I’m in a very safe space, and I feel like I’m talking to someone who really understands. - P5

I had one patient who was Sudanese. He had been discharged from the service, and we connected instantly. He didn’t mind that I was a graduate nurse who was doing my first rotation or anything of that sort. You could see that connection. He would reach out to me to ask me how I was feeling, even when I didn’t show up for work, like I had a day off or something; he would ask nurses about me. . .. - P6.

An aspect of cultural sensitivity highlighted by some participants was language. For example, P6 considered that language, and particularly the African accent, a significant barrier to mental health help-seeking. According to the participant: ‘You talk about language as well. . .As a nurse myself, you speak to someone, a Caucasian, and the person is like, “Sorry, I don’t get what you’re saying. . .” So, you have to slow down the rate of your speech or keep repeating yourself’. The participant thought that African migrants with mental health issues would not want to go where they would keep repeating themselves to be understood:

Imagine if it was reversed. An African person who is mentally ill, or living with mental illness, turns up in your service, and she’s trying to tell you things. . .and because you’re making her repeat herself more than she could do, but then she has to tell you because she’s come there, and that’s another stress. . .People look at all those things and like, 'no, I don’t want to go.’

Overall, participants view cultural insensitivity as a significant obstacle to mental health help-seeking by African migrants as it may cause them to feel that their culture, beliefs and identity are being dismissed dismissal. Some participants also believed that a lack of appreciation of African migrants’ language challenges or African accents could make them feel misunderstood, leading to hesitance in seeking mental health care.

### Cost of Mental Health Services

Most participants felt that healthcare cost in Australia was a significant deterrent to African migrants’ utilization of mental health services. They believed that the cost of healthcare was higher in Australia than in many other places, thus increasing the barriers to mental health help-seeking for African migrants, especially those without public-funded Medicare. The views of the participants are highlighted below:

. . . For some of us who do not have Medicare, we pay through private insurance, and in some cases, most of the GPs around here and most of the medical centres around here do not accept private healthcare cards. They ask you to pay out of pocket and go and remit your money—and go and reclaim your money later. This is a very big barrier for us. If you are going to access care, that may involve running some investigations; in some cases, you may need to pay about $300, and as you’re paying out of pocket, taking out $300 from your pocket is not easy. P-8

Mental health services in Australia are not cheap. The reason why people don’t always go is because they must pay for it. It’s not free. I think that’s one of the reasons why most people don’t, because you’re not going to solve a problem by creating one. I am going to see mental health, and at the end of the day, I’m not going to have money to live. – P10

The other thing is also that it’s not cheap. I think it’s just access because you don’t know the dosage necessary from the beginning. . . With these mental health issues, often you don’t know how many times you will need to see the therapist. It can be prohibitive. . .If you go to a psychologist, you get a mental health plan and rebate. I can’t remember how much - $190. . . If I send $190 home, it’ll be school fees for all three terms in the year for a child, for example. Competing priorities. - P.12

Clearly, many of the participants reasoned that the high cost of healthcare in Australia was a significant barrier to African migrants’ mental health help-seeking as it might create financial burdens and impact their ability to afford essential treatments.

## Discussion of Findings

Our study provides qualitative insight into the perceived barriers to mental health help-seeking among African migrants in Australia. The findings of this study have highlighted five key barriers to mental health help-seeking among African migrants in Australia. Participants believed religion and poor knowledge about mental illness were obstacles to mental health help-seeking. They also considered cultural beliefs, fear of stigma as well as the high cost of healthcare in Australia as major barriers to mental health help-seeking among African migrants.

In this study, many African migrants were more inclined to seek support for mental health issues from religious figures such as priests instead of seeking professional help, suggesting that African migrants considered faith healing is a significant pathway to mental healthcare. This confidence in religious figures reveals a broader cultural propensity to view mental health issues through a spiritual lens instead of a medical one. Pathway to care has been defined as ‘the sequence of contacts with individuals and organizations prompted by the distressed person’s efforts, and those of his or her significant others, to seek help’ (Rogier and Dharma, 1993, as cited in Lilford et al., 2020). It is common in Africa for individuals experiencing mental health challenges to seek help from traditional and religious faith healers initially. According to a Ghanaian study, faith and traditional healing pathways are utilized by individuals with mental health challenges as a preliminary source of cultural assessment before seeking professional help (Badu et al., 2019). Some participants, however, linked the reliance on religious intervention to a poor understanding of mental health and believed that many individuals lacked access to accurate mental health information or the available treatment options. Thus, they turned to religious leaders for support out of ignorance or misconception. Relying on religious leaders for mental health help is not peculiar to African migrants in Australia. For instance, pastors in Black churches in the United States serve as both spiritual advisors and mental health counselors to their members (Campbell & Winchester, 2020). In several low- and middle-income countries, faith healing is used in tandem with conventional treatment for many health problems, including mental illness (Kpobi & Swartz, 2018). Religion, in its many forms, has long been recognized as a significant source of succor and encouragement in times of difficulty (Schnabel & Schieman, 2021). It is widely acknowledged that holding religious beliefs and membership in faith communities can enhance the mental wellbeing of migrants (Copping et al., 2010; Mitha & Adatia, 2016; Russo et al., 2015). In a study of depression among Black immigrant women in Ontario, Canada, Schreiber et al. (1998) found that religion and spirituality played a significant role in accepting and managing depression, with participants interpreting depression from a religious context. Likewise, Said et al. (2021) investigated the barriers to accessing mental health services among Somali-Australian women and found that religion was perceived to be a barrier to help-seeking.

This finding, therefore, suggests that African migrants in Australia may not be receiving adequate mental health care, as relying solely on spiritual support may not sufficiently address underlying psychological problems, potentially stretching suffering or aggravating symptoms. Nevertheless, addressing this issue will require education and cooperation between mental health professionals and religious leaders. Raising awareness about mental health in the African population in Australia will help dispel misconceptions and encourage people to seek appropriate care.

Poor knowledge about mental illness can have a significant impact on help-seeking behavior, creating a gap that deters individuals with mental health challenges from seeking timely and appropriate help (Osman et al., 2022). Mental health knowledge entails ‘. . .being capable of identifying mental health problems, understanding risk factors and causes, professional help available, attitudes that promote recognition, and appropriate mental health help-seeking behaviors’ (Tesfaye et al., 2021, p. 2). Participants in our study believed that many African migrants in Australia had limited understanding of mental illness and its symptoms, often not knowing when to seek help from qualified professionals or reach out to support groups, including family members and friends.

This finding agrees with previous studies that explored barriers to mental health help-seeking among African migrants (Fauk et al., 2021; Henderson & Kendall, 2011; McCann et al., 2016; Pettersen & Debesay, 2023). Poor knowledge about mental illnesses can reinforce prejudices and misconceptions, engendering an environment where people may feel ashamed or reluctant to disclose their struggles and reach out for help (National Academies of Sciences, Engineering, and Medicine, 2016). Improving mental health literacy is one way to encourage early help-seeking for mental illness. Knowledge of mental health, including understanding different conditions, symptoms and available treatments, can positively influence behaviors toward help-seeking. Research has also established a nexus between knowledge of mental health and help-seeking behavior (Fung et al., 2021; Ratnayake & Hyde, 2019; Tesfaye et al., 2021).

Fear of stigma was identified as a significant barrier to mental health help-seeking among African migrants in Australia. Stigma is a mark of shame or disgrace that causes others to view a person or social group differently (Byrne, 2000). Individuals with mental health problems often suffer stigma, fear, discrimination, and rejection in the broader community (Link & Phelan, 2001), reinforcing cultural beliefs that associate mental health problems with personal weakness. Stigma produces a perversive atmosphere of shame and secrecy, whereby people experiencing mental health challenges may fear being judged or discriminated against if they disclose their struggles (Ussher et al., 2017). A Lancet review of studies on stigma reported that while the public might accept the medical or genetic nature of mental health illness and the need for treatment, several people still had negative views of individuals with mental illness (Thornicroft et al., 2016). Participants in our study noted that it was common within the African community to label individuals with mental health problems ‘mad’ or ‘crazy’, a situation that further perpetuates the reluctance to acknowledge and seek help for mental health problems. This indicates that both self-stigma (the internalization of shame, prejudices, and negative attitudes due to mental illness) and public stigma (the negative or discriminatory attitudes people in society have about mental illness) were hindrances to help-seeking. This finding is consistent with other studies that examined barriers to mental health help-seeking among African migrants (Boukpessi et al., 2023; McCann et al., 2017; Ogueji & Okoloba, 2022; Pettersen & Debesay, 2023; Said et al., 2021). Participants in our study also highlighted how cultural beliefs discourage help-seeking. Indeed, one participant noted that seeking help for mental illness was considered a sign of weakness in their culture.

It was not surprising that our participants highlighted cultural beliefs as a barrier to mental health help-seeking. Cultural beliefs are pivotal in shaping attitudes and perceptions about mental health in every society (Jang et al., 2007). These beliefs determine how people understand, interpret, and respond to issues of mental health, influencing help-seeking behaviors, choice of treatment, and social support networks. There is a surfeit of evidence in the literature that the perception of health and illness varies across cultures (Biswas et al., 2016; Fernando, 2015; Gopalkrishnan, 2018). In many cultures, mental health is entangled with religious, spiritual, and communal frameworks, which can both facilitate or deter mental health outcomes. Cultural norms and values determine what may be considered normal or abnormal behavior. For instance, in some cultures, it is viewed as a sign of weakness or personal failure to express distress or emotional suffering, causing people to suppress or deny their symptoms (Bennett et al., 2023). However, in other cultures, mental illness may be associated with spiritual causes or ancestral influences, impacting perceptions of treatment and recovery (Subu et al., 2022). This finding further underscores the need for socially inclusive and culturally sensitive mental health policies that recognize and accommodate the diverse cultural backgrounds of migrants. Culturally focused services will produce better outcomes for CALD communities and enable equitable access to programs and services (Mental Health in Multicultural Australia, 2014). Such services can facilitate participatory community-led mental health awareness campaigns that will help address cultural beliefs that encourage the stigmatization of mental illness, engender open dialog, and promote help-seeking behaviors (Apers et al., 2023). Utilizing tools like the cultural formulation interview (CFI) in assessing migrant clients can also improve culturally sensitive diagnosis and treatment. An interview protocol used for collecting essential data to improve culturally sensitive diagnosis and treatment, the CFI focuses on the patient’s perspective and social context during diagnostic evaluation (Jarvis et al., 2020). It allows patients to describe their experiences colloquially, not necessarily through biomedical terms or concepts (Jarvis et al., 2020). The CFI seeks to improve culturally sensitive diagnosis and treatment by focusing on the patient’s perspective and sociocultural context. In addition to guiding mental health professionals to explore how patients’ cultural background shapes their perceptions of mental health (La Roche & Bloom, 2018), the CFI makes it easy for professionals to identify idioms of distress – those specific ways in which members of sociocultural groups express affliction or distress (Hinton & Lewis-Fernández, 2010).

Another barrier from our study was poor service delivery, which manifested mainly in cultural insensitivity. Many participants said they would prefer to see mental health professionals from similar cultural backgrounds or at least those who would respect their cultural values, beliefs, and customs. It could be inferred from the participants’ observation that poor service delivery was causing impairments to their health. Some participants who suffered loss lamented not being supported to grief the way they would have loved to, thus casting doubt on their readiness to utilize the services of grief counselors in Australia or recommend the same to other African migrants. Other participants described instances of racism, noting, for example, how some Caucasian mental health workers appeared to mock the accents of African migrants seeking help. These are consistent with previous studies in Australia and overseas highlighting how racism, cultural insensitivity and, more broadly, poor service delivery discourage African migrants from seeking professional help for their mental health challenges (Bassey & Zaka, 2024; Boukpessi et al., 2021; McCann et al., 2016; Ogueji & Okoloba, 2022; Wamwayi et al., 2019). Migrants may encounter communication problems, misconstruing of their experiences, and feelings of marginalization within the healthcare system when mental health services are not culturally sensitive (Kirmayer et al., 2011). Cultural insensitivity can take different forms, including poor communication skills, a lack of awareness of cultural beliefs, or an inability to identify the effect of migration-related stressors associated with mental health (Bustamante et al., 2017; Fernando, 2004). These factors aid poor service delivery and discourage African migrants from trusting the healthcare system and seeking formal help. Addressing the barrier of cultural insensitivity and poor service delivery requires structural changes within healthcare systems. For instance, mental health professionals might be required to undergo cultural humility training so that they can learn to reflect on their own beliefs and biases and how they might impact their patients (Lekas et al., 2020).

Our study also identified the financial costs of obtaining professional mental health support as a deterrent to help-seeking, particularly for those without access to public-funded Medicare. This problem is exacerbated by service provider’s refusal to accept some private health insurance, requiring patients to pay out of pocket and reclaim their expenses later. The requirement to pay a significant amount upfront for consultations, investigations, or therapy sessions can be both arduous and off-putting., especially for many African migrants who are already constrained financially. Additionally, the stress of negotiating the healthcare system, including understanding eligibility criteria for public-funded mental health services, exacerbates the situation for a group of people already struggling with stigma and cultural and language barriers. Nonetheless, the impact of the financial barrier transcends the cost of consultation and treatment. Our study highlighted the dilemma African migrants face in allocating scarce resources between healthcare expenses and competing priorities such as education, housing and support for family members in their home countries. This underlines the wider socioeconomic disparities that determine access to mental health care within the African population in Australia. The cost of mental health services is recognized as a barrier to help-seeking (Rowan et al., 2013) and has also been highlighted in other studies examining barriers to mental health help-seeking among African migrants (McCann et al., 2016; Sheikh-Mohammed et al., 2006). Eliminating or lowering the barriers to mental health care is critical for enhancing the health and wellbeing of African migrants in Australia and ensuring that financial challenges cannot deter them from accessing professional mental health services. Addressing the financial barrier to help-seeking requires extensive policy interventions targeted at improving affordability and accessibility for individuals, irrespective of their socioeconomic and immigration status. This may entail widening public-funded mental health services and reducing out-of-pocket expenses for socioeconomically marginalized individuals.

## Conclusions

The present study has shed light on the barriers to mental health help-seeking among African migrants in Australia. Participants identified religion and limited knowledge about mental illness as significant deterrents to seeking mental health support. Additionally, cultural beliefs, fear of stigma, and the high cost of healthcare in Australia emerged as significant obstacles. These findings have profound implications for migrants, mental health care providers, policymakers, and governments. Importantly, our research underscores the positive contribution of religion and religious leaders to the mental wellbeing of many African migrants in Australia. This underlines the importance of fostering dialog and partnerships between mental health services and religious organizations. Governments can also encourage and support religious leaders to undergo formal mental health and counseling training through grants and scholarships, thus ensuring sensitive care for African migrants with mental health concerns.

Furthermore, findings emphasize the critical need for culturally competent mental health services tailored to the beliefs, values, religion, and experiences of African and other migrant communities. Given the strong attachment of many African migrants in Australia to their cultural and religious beliefs, such services are essential for practical support and intervention. While this study is valuable, it has limitations. Majority of the participants identified as Christians, which meant the study could not explore how other religious beliefs and practices influence mental health help-seeking behaviors. Also, the relatively small number of participants raises concerns about generalizability. However, as a qualitative study, the focus was on gaining insights into our participants’ experiences within the specific context of our study. While strict generalization may not be feasible, the present findings offer valuable insights that may resonate with similar populations in other settings. Furthermore, given the heterogeneity of the African population in Australia, including its demographic diversity, future research could explore how regional differences, socioeconomic status, migration duration, and acculturation influence perspectives and experiences. Finally, the findings of this study contribute to the growing body of literature on migrants’ mental health by documenting the perceived barriers to mental health help-seeking among African migrants in Australia. There is a clear need for further research to explore the role of religion in the mental health of African migrants, thereby fostering a deeper understanding and more effective support mechanisms for this vulnerable population.

## Notes

1. African-born includes those born in both Sub-Saharan Africa and North Africa.
